# Comparison of GLS via echocardiography and tissue tracking by Cardiac Magnetic Resonance imaging

**DOI:** 10.1186/1532-429X-17-S1-P77

**Published:** 2015-02-03

**Authors:** John-Paul Tantiongco, Suchi Grover, Rebecca Perry, Craig Bradbrook, Darryl Leong, Joseph Selvanayagam

**Affiliations:** 1 Flinders Medical Centre, Bedford Park, SA, Australia; 2 Flinders University, Bedford Park, SA, Australia

## Background

Global longitudinal strain (GLS), has shown utility in detecting early subclinical LV dysfunction and has demonstrated significant efficacy in evaluating patients for chemotherapy cardiotoxicity. However, echocardiography is often reliant on operator experience and adequate quality windows for assessment. CMR is not subject to limitations of poor image quality due to its superior endocardial definition. We evaluated CMR derived strain by implementing a tissue tracking algorithm and compared it to GLS by 2D echocardiography in a prospective study of chemotherapy patients.

## Methods

10 patients with breast cancer and receiving cardiotoxic chemotherapy (anthracycline and/or trastuzumab) underwent concurrent transthoracic echocardiography (TTE) for GLS and CMR to assess myocardial function and strain (tissue tracking). 2D TTE was performed by an experienced operator using established techniques (speckle tracking). A novel CMR tissue tracking program (Circle Cardiovascular Imaging Inc) was performed by delineating endocardial and epicardial contours on cine images during diastole in 3 long axis views (VLA, HLA and LVOT) and automated algorithm was applied to derive strain values for each image. These were averaged, to derive global longitudinal strain by CMR and comparison with GLS by TTE was done using Bland-Altman analysis.

## Results

**Figure 1:**
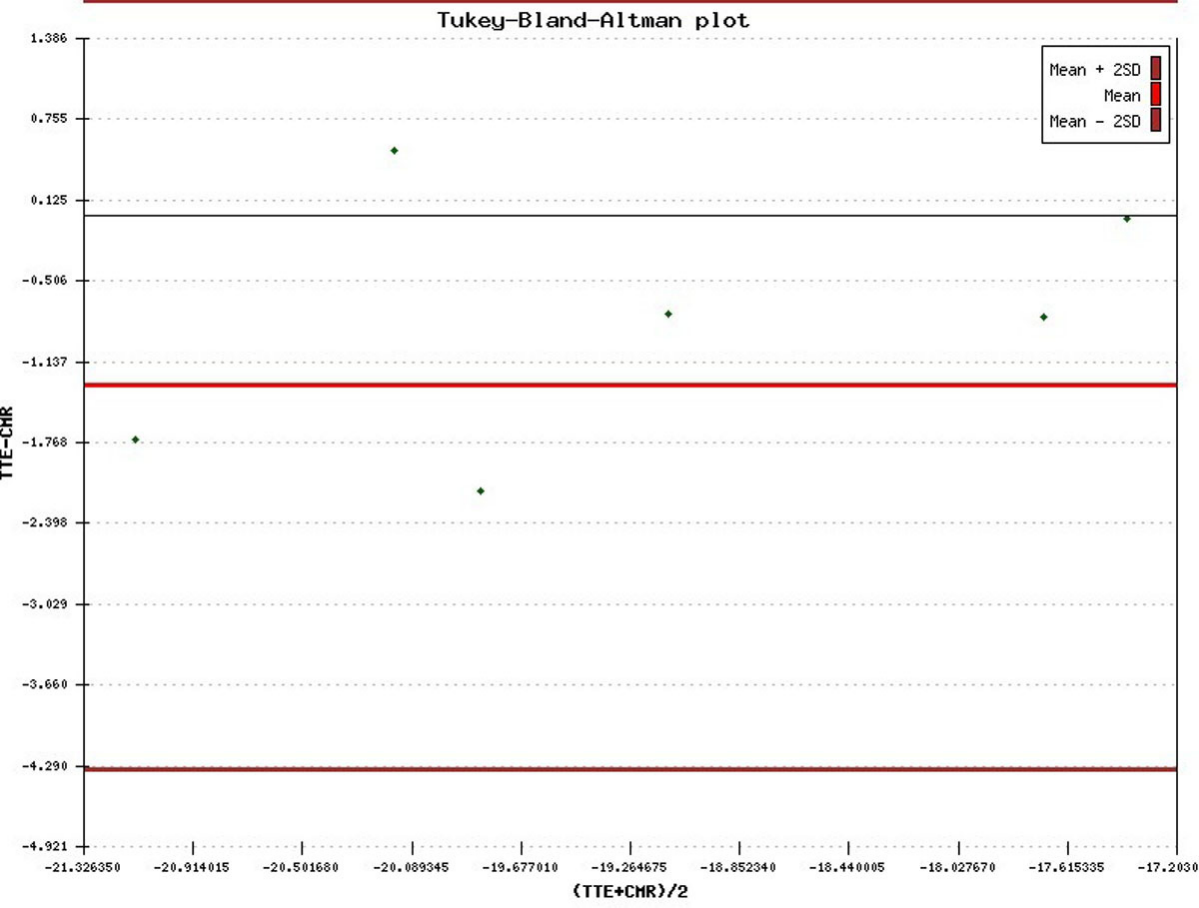


The mean age of the women was 46 ± 9.3 (SD) years. Myocardial function was preserved in all patients with mean ejection fraction 71 ± 3% (SD). Bland-Altman analysis of TTE GLS versus CMR GLS reveals a bias of -1.322 ± 1.50 (SD) which is within the 95% limits of agreement (-4.322, 1.678). (See Figure 1)

## Conclusions

CMR derived strain may be used interchangeably with TTE derived GLS. This needs to validated in a larger cohort with a wider range of myocardial dysfunction.

## Funding

N/A.

